# Accelerated Full-Thickness Wound Healing by a Topical Ointment Formulated with *Lobelia alsinoides* Lam. Ethanolic Extract

**DOI:** 10.3390/ijms262110663

**Published:** 2025-11-01

**Authors:** Rex Jeya Rajkumar Samdavid Thanapaul, Sreeraj K. Manikandan, Mosae Selvakumar Paulraj, M. S. A. Muthukumar Nadar

**Affiliations:** 1 Division of Biotechnology, Karunya Institute of Technology and Sciences, Coimbatore 641114, Tamil Nadu, India; haiiamrex@gmail.com (R.J.R.S.T.); sreerajkm94@gmail.com (S.K.M.); 2 Environmental Sciences Program, Green Bangle Movement, Asian University for Women, Chittagong 4000, Bangladesh; p.selvakumar@auw.edu.bd

**Keywords:** *Lobelia alsinoides* Lam., wound healing, antimicrobial, antioxidant, ointment, phytotherapeutic, toxicity

## Abstract

Chronic wounds present a major clinical challenge, often aggravated by infection and rising antimicrobial resistance. This study investigated the wound-healing efficacy of *Lobelia alsinoides* Lam., an ethnomedicinal herb, formulated as a topical ointment containing its ethanolic extract (LT). Phytochemical profiling identified high levels of phenolics, terpenoids, and tannins, while in vitro assays demonstrated strong antioxidant, broad-spectrum antimicrobial, and cytocompatible properties. Wound-healing potential was evaluated using excision and incision wound models in rats treated with 5% or 10% LT ointments, with Silverex™ as the reference standard. The 10% LT formulation significantly outperformed Silverex™, accelerating wound contraction (99.33 ± 0.55% by Day 16), shortening epithelialization time (16.1 ± 0.8 days), and enhancing tensile strength (837.36 ± 16.37 g; *p* < 0.001). Biochemical and histological analyses confirmed improved collagen deposition, extracellular matrix remodeling, and angiogenesis, without hepatic or renal toxicity. Overall, LT exhibited statistically superior wound-healing efficacy compared with Silverex™, supporting its potential as a safe, affordable, and sustainable phytotherapeutic alternative. These findings provide strong scientific validation for *L. alsinoides* as an evidence-based herbal candidate for integration into modern wound care, with future studies warranted to establish mechanistic and clinical efficacy in chronic and infected wounds.

## 1. Introduction

Wound healing is a complex physiological process comprising overlapping stages of hemostasis, inflammation, proliferation, and remodeling, which collectively restore the continuity and functionality of damaged skin [1,2,3]. Despite significant advances in surgical techniques, wound dressings, and pharmacological agents, chronic and non-healing wounds remain a major global health concern, affecting more than 40 million people worldwide and imposing substantial physical, emotional, and economic burdens [4,5]. These wounds, often precipitated by infection, oxidative stress, diabetes, or vascular insufficiency, pose particular challenges in aging populations and resource-limited settings [6]. The rise of antimicrobial resistance (AMR) further complicates treatment by diminishing the efficacy of conventional antibiotics and antiseptics, thereby emphasizing the urgent need for safer and more sustainable wound care solutions [7,8].

Medicinal plants have provided therapeutic alternatives for treating skin injuries for centuries and remain rich sources of bioactive compounds [9,10,11,12]. Secondary metabolites such as flavonoids, phenolics, terpenoids, tannins, and alkaloids are well recognized for their antioxidant, antimicrobial, anti-inflammatory, and tissue-regenerative properties [12,13,14]. Mechanistically, these phytochemicals modulate key cellular pathways involved in wound repair, including TGF-β/Smad, VEGF, NF-κB, and Nrf2 signaling [15,16]. However, most traditional herbal preparations lack rigorous scientific validation, and only a few have undergone comprehensive preclinical evaluation integrating phytochemical profiling, multi-assay in vitro analyses, and functional validation in standardized full-thickness animal wound models [17,18]. Limitations of currently available herbal agents include inconsistent efficacy, lack of standardization, and insufficient mechanistic or comparative studies, particularly regarding extracellular matrix (ECM) remodeling and chronic wound healing.

**Figure 1:**
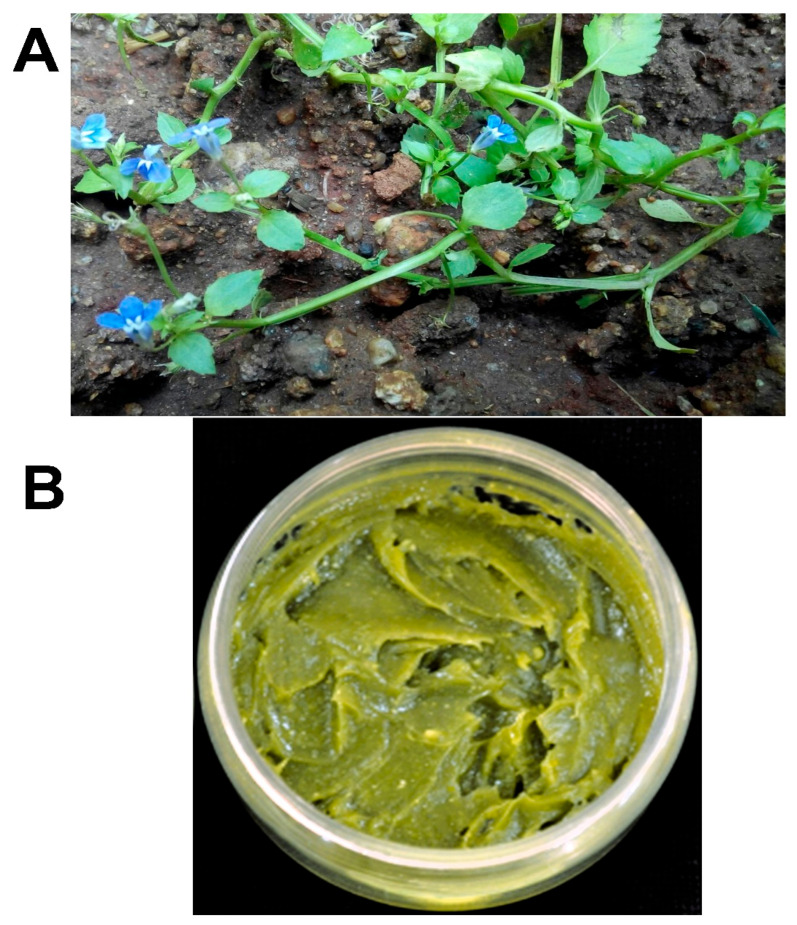
*Lobelia alsinoides* Lam. and formulation of the ethanolic extract ointment. (**A**) Whole plant of *L. alsinoides* Lam. (**B**) Topical ointment prepared with *L. alsinoides* ethanolic extract (10% *w*/*w*) incorporated into a simple emulsifying base for wound-healing evaluation.

*Lobelia alsinoides* Lam. (syn. *Lobelia trigona* Roxb. (LT) (Figure 1A)) is a small herbaceous plant native to India and Southeast Asia [19,20,21]. Valued in ethnomedicine as both a wild edible and a traditional remedy for inflammatory conditions, its culinary use also indicates a favorable safety profile [22]. Pharmacognostic research has established its taxonomic identity and standardization parameters, while preclinical studies have demonstrated hepatoprotective, antimicrobial, and antioxidant properties, collectively supporting its therapeutic potential [19,23,24,25].

Previous investigations, including our own, have identified diverse flavonoids, phenolics, and alkaloids in *L. alsinoides*, with notable antioxidant and burn wound-healing activities [23,26,27]. We previously demonstrated that the ethanolic extract of *L. alsinoides* accelerates the healing of third-degree thermal burns [26], primarily by promoting epithelial regeneration and collagen maturation. Our subsequent work using *L. trigona* Roxb-derived nanomedicine [28] further highlighted nanoscale enhancement of antioxidant and antimicrobial efficacy, while our recent phytochemical profiling of *L. alsinoides* [23] provided detailed compositional and cytotoxic validation of its bioactive constituents.

Despite these important advances, most available studies, including our own, have been limited to acute or burn wound models and have not comprehensively assessed full-thickness excision or incision repair, ECM remodeling, or angiogenic responses. Unlike previous investigations that primarily focused on superficial wounds, the present study fills a critical research gap by conducting a holistic evaluation of *L. alsinoides* in standardized full-thickness excision and incision models. By integrating detailed phytochemical quantification, antioxidant and antimicrobial assessments, cytocompatibility studies using human keratinocytes, and biochemical and histological analyses of ECM remodeling and angiogenesis, this work represents the first comprehensive preclinical validation of *L. alsinoides* mediated ointment for deep-tissue wound repair. This approach not only confirms the therapeutic reproducibility of *L. alsinoides* across wound types but also establishes its translational relevance as a scientifically standardized phytopharmaceutical alternative to conventional silver-based formulations.

The present investigation advances this framework by incorporating (i) quantitative phytochemical profiling, (ii) antioxidant and antimicrobial evaluation, (iii) in vitro cytocompatibility assessment using HaCaT keratinocytes, and (iv) in vivo analyses of ECM remodeling and angiogenic biomarkers in standardized full-thickness rat wound models. Moreover, the inclusion of both 5% and 10% ointment concentrations, previously validated in burn models [26], enables direct cross-study comparison and establishes reproducibility across wound contexts. This multidimensional and comparative design provides the first holistic preclinical validation of *L. alsinoides* ointment for deep-tissue wound healing. The therapeutic activity of *L. alsinoides* is hypothesized to derive from its antioxidant ability to neutralize reactive oxygen species, its broad-spectrum antimicrobial effect to reduce infection, and its capacity to promote fibroblast migration, angiogenesis, and collagen deposition.

In this study, we systematically formulated and evaluated a topical ointment containing *L. alsinoides* ethanolic extract (Figure 1B). Our comprehensive experimental strategy encompassed phytochemical profiling, antioxidant and antimicrobial assays, cytotoxicity testing, and detailed assessments of wound healing in full-thickness excision and incision models in rats. These evaluations included measurements of re-epithelialization, tensile strength, angiogenesis, collagen organization, and ECM biomarker dynamics. Comparative validation with a standard silver-based ointment (Silverex™) was also performed, alongside discussions of formulation stability, safety, and regulatory implications.

By integrating ethnomedicinal insights with rigorous pharmacological assessment, this study aims to validate *L. alsinoides* as a safe, effective, and accessible therapeutic candidate for wound management, addressing major limitations of current therapies and supporting its translational potential in regions where standard interventions remain inadequate or unavailable.

## 2. Results

### 2.1. Quantitative Phytochemical Profiling

The ethanolic extract of *L. alsinoides* was quantitatively analyzed to determine the concentrations of key bioactive secondary metabolites. As shown in Supplementary Figure S1, the extract exhibited a high content of total phenolics (42.3 ± 1.2 mg gallic acid equivalents (GAE)/g, *p* < 0.001), followed by total terpenoids (25.6 ± 0.9 mg ursolic acid equivalents (UAE)/g, *p* < 0.001) and total tannins (15.8 ± 0.7 mg gallic acid equivalents (GAE)/g, *p* < 0.001). Moderate quantities were recorded for alkaloids (8.2 ± 0.5 mg atropine equivalents (AE)/g, *p* < 0.01) and flavonoids (7.1 ± 0.4 mg rutin equivalents (RE)/g, *p* < 0.01), while the total saponin content was comparatively lower (2.5 ± 0.3 mg ginsenoside equivalents (GSE)/g, *p* < 0.01). These results indicate that *L. alsinoides* is predominantly enriched in phenolic and terpenoid constituents.

### 2.2. Antimicrobial Activity of Lobelia alsinoides Lam Ethanolic Extract

The ethanolic extract of *L. alsinoides* exhibited broad-spectrum, dose-dependent antibacterial and antifungal activity against a comprehensive panel of clinically relevant microorganisms, including both Gram-positive and Gram-negative bacteria as well as pathogenic fungi (Table 1; Supplementary Figure S2). The antimicrobial efficacy increased proportionally with extract concentration (25, 50, and 100 µg/mL), confirming a strong concentration-response relationship.

Among the Gram-positive bacteria, *Bacillus subtilis*, *Streptococcus pneumoniae*, and methicillin-resistant *Staphylococcus aureus* (MRSA) were the most susceptible strains, showing inhibition zones of 17.67 ± 1.92 mm, 17.67 ± 1.83 mm, and 18.33 ± 1.38 mm, respectively, at 100 µg/mL. *S. aureus* and *S. epidermidis* also demonstrated strong inhibition zones (17.67 ± 1.89 mm and 18.00 ± 1.92 mm, respectively), with MIC values ranging from 10 to 12 µg/mL and MBCs of 25 µg/mL, indicating a bactericidal mode of action. Notably, MRSA was equally sensitive to the extract as the methicillin-susceptible *S. aureus* strain, highlighting the potential of *L. alsinoides* extract against resistant bacterial phenotypes. By comparison, the probiotic *Lactobacillus acidophilus* exhibited a smaller inhibition zone (16.67 ± 1.88 mm) and a slightly higher MIC (15 µg/mL), indicating selective antibacterial activity favoring pathogenic over commensal species.

Within the Gram-negative bacterial panel, the *L. alsinoides* extract demonstrated potent and consistent inhibition across multiple strains. *Klebsiella pneumoniae* and *Salmonella paratyphi* exhibited the largest inhibition zones (19.00 ± 1.64 mm and 18.67 ± 1.72 mm, respectively) at 100 µg/mL, with corresponding MICs of 12 µg/mL and 10 µg/mL, and MBCs of 25 µg/mL. The multidrug-resistant *Acinetobacter baumannii* was also highly susceptible, with a zone of inhibition of 17.67 ± 1.55 mm and a MIC of 10 µg/mL, emphasizing the extract’s therapeutic promise against resistant pathogens. Other Gram-negative bacteria, including *Escherichia coli*, *Pseudomonas aeruginosa*, *Serratia marcescens*, and *Shigella sonnei*, showed moderate to strong susceptibility, with inhibition zones ranging between 16.67 and 17.66 mm, MIC values of 12–18 µg/mL, and MBCs of 26–28 µg/mL, further demonstrating the broad-spectrum antibacterial potency of the *L. alsinoides* extract.

The antifungal activity of the *L. alsinoides* extract was similarly notable, displaying a clear concentration-dependent inhibitory pattern. *Aspergillus fumigatus* was the most sensitive fungal strain, with a maximum inhibition zone of 19.11 ± 1.78 mm and low MIC and MFC values of 10 µg/mL and 25 µg/mL, respectively. Other species, including *Candida albicans*, *C. tropicalis*, and *Trichophyton rubrum*, exhibited strong susceptibility, with inhibition zones ranging from 18.10 to 18.66 mm, MICs of 12–14 µg/mL, and MFCs of 28 µg/mL. *Aspergillus flavus* and *A. niger* demonstrated moderate inhibition zones (17.33–17.58 mm), with MICs of 12–15 µg/mL and MFCs of 26–28 µg/mL.

MIC and MBC/MFC values were determined as the lowest concentrations that completely inhibited visible microbial growth compared to untreated controls and were validated spectrophotometrically by measuring optical density at 600 nm for bacterial cultures and 530 nm for fungal cultures. Concentrations achieving ≥90% reduction in culture turbidity were designated as MICs, while MBC/MFC values were confirmed by subculturing onto fresh agar plates, where ≥99.9% microbial kill indicated bactericidal or fungicidal activity. Statistical analysis revealed highly significant differences (*p* < 0.001) between MIC and MBC/MFC values across all tested bacterial and fungal species, confirming the dose-dependent antimicrobial efficacy of the *L. alsinoides* extract.

### 2.3. Antioxidant Activity of Lobelia alsinoides Lam Ethanolic Extract

**Figure 2:**
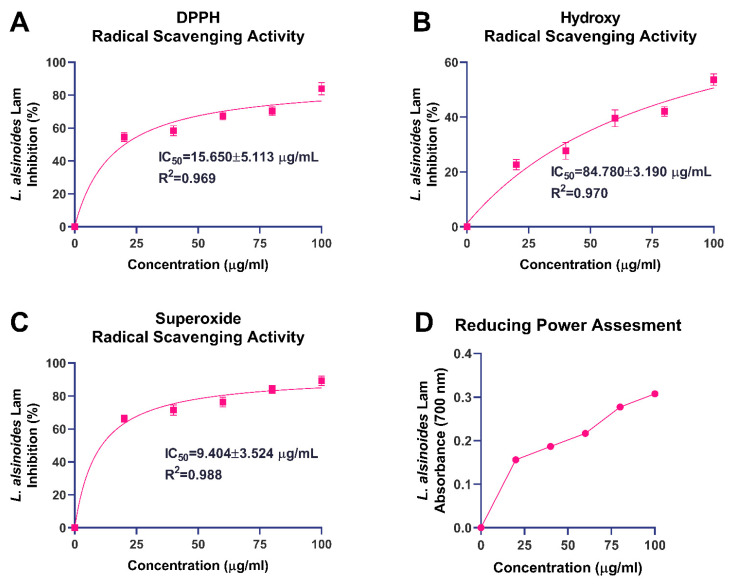
Antioxidant activity of the ethanolic extract of *Lobelia alsinoides* Lam. (**A**) DPPH radical scavenging activity showing a concentration-dependent inhibition with an IC_50_ value of 15.65 ± 5.11 µg/mL (R^2^ = 0.969). (**B**) Hydroxyl radical scavenging activity demonstrating moderate, dose-dependent inhibition with an IC_50_ value of 84.78 ± 3.19 µg/mL (R^2^ = 0.970). (**C**) Superoxide radical scavenging activity exhibiting strong inhibition with an IC_50_ value of 9.40 ± 3.52 µg/mL (R^2^ = 0.988), indicating potent superoxide-neutralizing capacity. (**D**) Reducing power assay showing a progressive, concentration-dependent increase in absorbance at 700 nm, reflecting enhanced Fe^3+^ to Fe^2+^ conversion and strong electron-donating potential. Square markers represent concentration versus percentage of inhibition in radical scavenging assays (**A**–**C**), while circle markers represent concentration versus absorbance in the reducing power assay (**D**). Data are expressed as mean ± SEM (*n* = 3). Abbreviation: IC_50_, half-maximal inhibitory concentration.

The antioxidant potential of the ethanolic extract of *L. alsinoides* was evaluated by targeting key reactive oxygen species (ROS), including DPPH, hydroxyl, and superoxide radicals, along with a reducing power assay (Figure 2). These complementary assays provided an integrated understanding of the extract’s electron-donating ability and free radical-neutralizing efficiency, both of which are critical for mitigating oxidative stress associated with tissue repair and regeneration. Nonlinear regression and goodness-of-fit parameters for all radical scavenging assays are summarized in Supplementary Table S1.

In the DPPH radical scavenging assay, the extract exhibited pronounced, concentration-dependent antioxidant activity (Figure 2A). The percentage inhibition increased steadily with rising concentrations, indicating effective quenching of DPPH radicals via hydrogen- or electron-transfer mechanisms. Nonlinear regression analysis yielded an IC_50_ of 15.65 ± 5.11 µg/mL with an excellent correlation coefficient (R^2^ = 0.969), demonstrating strong agreement between the experimental data and the fitted dose–response model. The hydroxyl radical scavenging assay showed a moderate yet consistent, concentration-dependent increase in inhibition (Figure 2B). The extract achieved approximately 55% inhibition at 100 µg/mL, with an IC_50_ of 84.78 ± 3.19 µg/mL and a high correlation coefficient (R^2^ = 0.970). Similarly, the extract exhibited marked superoxide radical scavenging activity (Figure 2C), showing a sharp, concentration-dependent increase that reached about 90% inhibition at 100 µg/mL. Nonlinear regression analysis produced an IC_50_ of 9.40 ± 3.52 µg/mL with an outstanding R^2^ value of 0.988, signifying an excellent model fit. The reducing power assay further supported the extract’s redox potential, revealing a steady, concentration-dependent increase in absorbance at 700 nm (Figure 2D). The absorbance rose from 0.156 ± 0.008 at 20 µg/mL to 0.308 ± 0.009 at 100 µg/mL, demonstrating enhanced Fe^3+^-to-Fe^2+^ reduction with increasing extract concentration.

### 2.4. Cytotoxicity and Wound Healing Bioactivity of Lobelia alsinoides Lam Ethanolic Extract in HaCaT Keratinocytes

The cytocompatibility of *L. alsinoides* ethanolic extract was evaluated using an MTT assay on human HaCaT keratinocyte cells to assess its safety and potential regenerative compatibility. As shown in Supplementary Figure S3, *L. alsinoides* exhibited a dose-dependent reduction in cell viability, with an IC_50_ value of 29.86 ± 6.34 µg/mL and a strong goodness of fit (R^2^ = 0.969), indicating robust model performance. The 95% confidence interval (CI) for the IC_50_ ranged from 20.10 to 46.26 µg/mL, confirming the precision of the fitted parameters. The extract maintained over 90% cell viability at concentrations below 20 µg/mL, suggesting minimal cytotoxicity at lower doses. These results demonstrate that *L. alsinoides* extract is well tolerated by human keratinocytes, supporting its biocompatibility and safety for subsequent in vitro and in vivo wound-healing applications.

To further establish a systemic safety margin, the *L. alsinoides* extract was subjected to a brine shrimp lethality assay, a widely used preliminary toxicity screen. No mortality was observed during the initial 6 h post-exposure, while LC_50_ values were determined as 1.6 mg/mL at 12 h and 0.75 mg/mL at 24 h (Supplementary Table S2). These values fall within the accepted non-toxic thresholds for natural products tested in *Artemia salina*, reinforcing the extract’s favorable safety profile and low lethality risk at pharmacologically relevant concentrations.

Beyond safety, the wound-healing potential of *L. alsinoides* was examined using an in vitro scratch assay on confluent HaCaT cell monolayers. Following the induction of a uniform scratch, cultures were treated with *L. alsinoides* extract at concentrations of 10, 50, and 100 µg/mL, and wound closure was monitored over 24 h (Supplementary Figure S4). The untreated control showed minimal spontaneous keratinocyte migration, whereas LT-treated groups displayed a clear dose-dependent enhancement in wound closure. Notably, treatment with 100 µg/mL *L. alsinoides* resulted in near-complete closure of the scratch area, 50 µg/mL produced substantial narrowing of the wound gap, and even the lowest concentration (10 µg/mL) significantly improved closure relative to baseline. These findings demonstrate that *L. alsinoides* extract promotes keratinocyte motility, a key process in re-epithelialization during the early stages of cutaneous wound repair.

### 2.5. In Vivo Safety and Wound Healing Efficacy of Lobelia alsinoides Lam Ethanolic Extract

A preliminary acute dermal toxicity study was conducted to evaluate the safety profile of *L. alsinoides* ethanolic extract prior to wound-healing experiments. Male Wistar rats were topically administered a limit dose of 2000 mg/kg body weight and observed for 14 days for any signs of systemic or local toxicity. As reported in Supplementary Table S2, no mortality, behavioral changes, dermatological reactions, or distress were observed in any subject. These findings suggest an LD_50_ greater than 2000 mg/kg, indicating a wide safety margin and excellent dermal tolerance.

**Figure 3:**
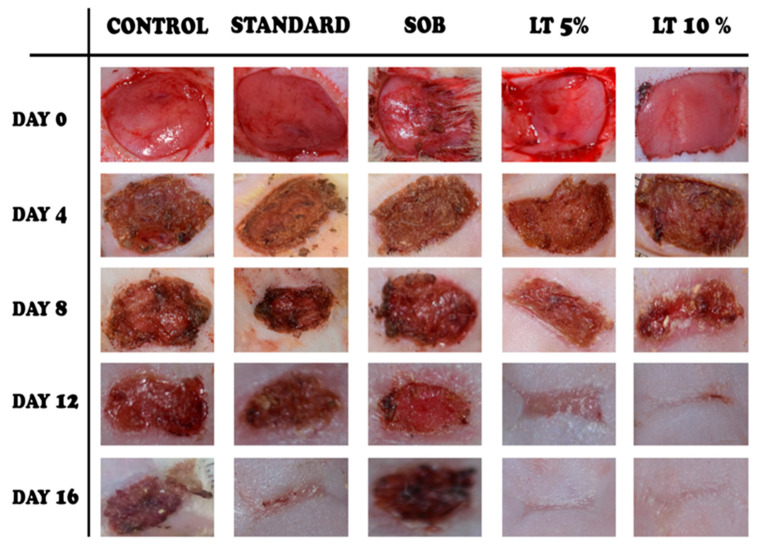
Macroscopic evaluation of excision wound healing in rats treated with *Lobelia alsinoides* Lam. (LT) ethanolic extract ointment. Representative wound photographs are shown for each treatment group: untreated control, standard reference (Silverex™), simple ointment base (SOB), 5% LT ointment, and 10% LT ointment, captured on Days 0, 4, 8, 12, and 16 post-wounding. The control and SOB groups exhibited delayed healing characterized by persistent scab formation and incomplete closure. The standard group showed progressive improvement, whereas LT-treated wounds demonstrated accelerated healing in a dose-dependent manner, with the 10% LT group achieving near-complete closure and re-epithelialization by Day 16.

The wound-healing efficacy of *L. alsinoides* extract ointment was assessed using a full-thickness excision wound model in rats. Five groups were studied: untreated control, simple ointment base (SOB), standard reference (Silverex™), and two *L. alsinoides* extract ointment formulations at 5% and 10% *w*/*w*. Wound areas were measured on Days 0, 4, 8, 12, and 16 (Figure 3), with all groups starting from comparable wound sizes. By Day 4, the 10% *L. alsinoides* extract ointment group showed early scab formation, smoother wound margins, and reduced exudation relative to the control and SOB groups, which exhibited persistent inflammation. Pronounced wound contraction and granulation tissue formation were evident in both *L. alsinoides* extract ointment-treated groups by Day 8, with the 10% formulation outperforming all others. Healing accelerated across *L. alsinoides* extract ointment-treated groups by Day 12, with the 10% group achieving near-complete epithelialization and absence of necrosis. By Day 16, complete wound closure and re-epithelialization were observed in the 10% *L. alsinoides* extract ointment group, which exhibited macroscopically superior wound appearance compared to Silverex™-treated animals. The 5% *L. alsinoides* extract treated group also demonstrated marked improvement, while the control and SOB groups retained partially open wounds.

**Figure 4:**
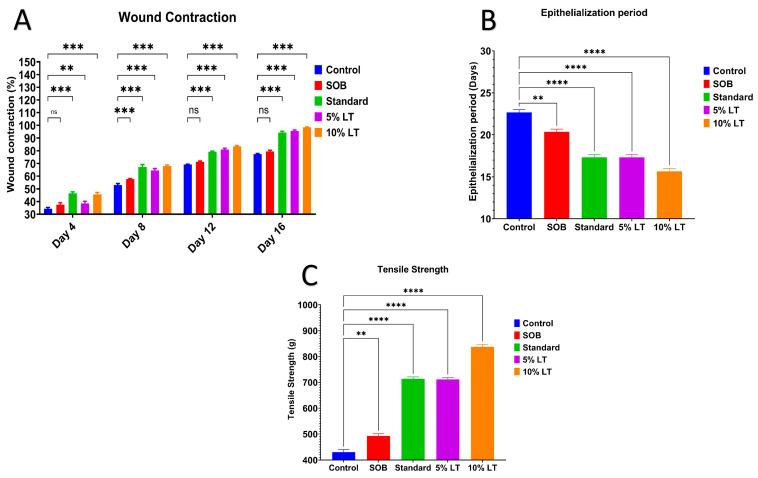
Quantitative evaluation of wound-healing parameters in rats treated with *Lobelia alsinoides* Lam. (LT) ethanolic extract ointments. (**A**) Percentage of wound contraction measured on Days 4, 8, 12, and 16 post-wounding. Both 5% LT and 10% LT groups exhibited significantly enhanced wound contraction compared with the control and SOB groups, with 10% LT showing the most pronounced effect. (**B**) Epithelialization period (days required for complete scab detachment without residual raw area). LT-treated groups demonstrated significantly shorter epithelialization times relative to the control and SOB groups, with the 10% LT group showing the fastest recovery. (**C**) Tensile strength of healed skin measured on Day 10 in the incision model. LT treatment, particularly at 10%, significantly increased tensile strength compared with the control, SOB, and standard Silverex™ groups, indicating superior dermal remodeling. Data are expressed as mean ± SEM (*n* = 6/group/experiment). Statistical analysis was performed using one-way or two-way ANOVA followed by Tukey’s multiple comparison post hoc test. ** *p* < 0.01, *** *p* < 0.001, **** *p* < 0.0001; ns, not significant.

Quantitative analysis corroborated these findings (Figure 4A). On Day 16, wound contraction was highest in the 10% *L. alsinoides* ointment-treated group (99.33 ± 0.55%), followed by 5% *L. alsinoides* ointment-treated (93.55 ± 0.24%) and Silverex™-treated (89.29 ± 0.63%) groups, with significantly lower values in the SOB (77.50 ± 0.67%) and control (70.35 ± 0.13%) groups. Statistical analysis revealed a highly significant treatment effect (F = 42.26, *p* < 0.001, R^2^ = 0.8879), confirming the superior wound contraction achieved by the 10% LT formulation compared with Silverex™ (*p* < 0.001). Tukey’s post hoc analysis showed significant improvements in wound contraction between Days 4 and 12 (*p* < 0.001), with healing stabilizing thereafter (Supplementary Table S3).

The epithelialization period, indicative of wound maturity, was significantly shortened in LT-treated groups. The 10% LT ointment-treated group exhibited the shortest epithelialization time (16.1 ± 0.8 days), followed by 5% LT ointment-treated (17.10 ± 1.0 days) and Silverex™-treated (17.91 ± 1.2 days) groups. By comparison, the SOB and control groups required 20.64 ± 0.9 and 22.74 ± 1.1 days, respectively, for complete scab detachment (Figure 4B). These data clearly indicate that the 10% LT ointment significantly accelerated epidermal regeneration relative to Silverex™.

Biomechanical recovery was evaluated by measuring the tensile strength of healed skin on Day 10 using an Instron tensiometer, further supporting these findings. The 10% LT ointment-treated group exhibited the highest tensile strength (837.36 ± 16.37 g), which was significantly greater than 5% LT ointment-treated (711.62 ± 12.90 g), Silverex™-treated (713 ± 14.59 g), SOB (480 ± 12.38 g), and control (430.37 ± 17.47 g) groups (*p* < 0.0001) (Figure 4C). These results confirm that 10% LT treatment enhanced dermal integrity and collagen cross-linking to a greater extent than the standard Silverex™ formulation.

Throughout the study, no adverse effects or abnormal behaviors were observed in any treatment group. Stable body weights further supported systemic safety following topical LT ointment application.

### 2.6. Biochemical Evaluation of Granulation Tissue

Biochemical analysis of granulation tissue provided compelling evidence that topical application of the *L. alsinoides* extract ointment markedly promotes ECM synthesis and remodeling, a key process for effective wound healing. Four critical ECM biomarkers, uronic acid, hexosamine, hydroxyproline, and total protein, were quantitatively measured in granulation tissues collected on Days 4, 8, 12, and 16 post-treatments. Across all markers, the 10% LT ointment-treated group consistently demonstrated the most pronounced and statistically significant increases, particularly during the proliferative and early remodeling phases, exceedingly even the standard Silverex™ group.

**Figure 5:**
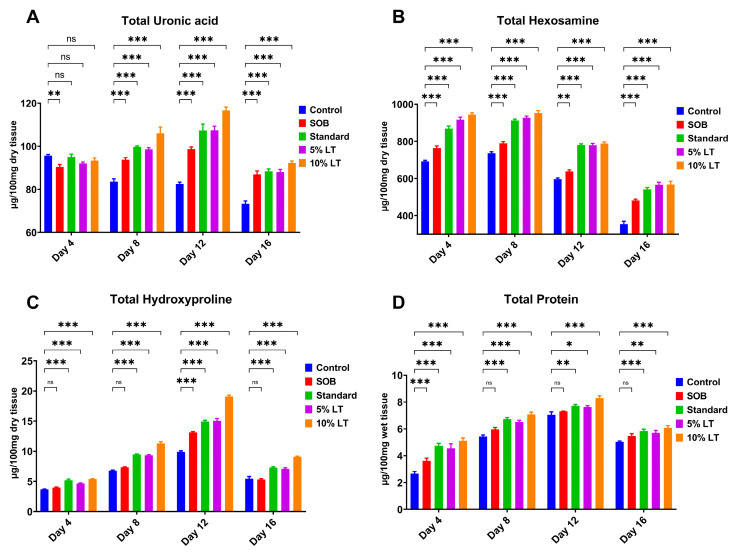
Biochemical analysis of extracellular matrix (ECM) components in granulation tissue during wound healing in rats treated with *Lobelia alsinoides* Lam. ethanolic extract ointments (LT). Tissue samples were collected on Days 4, 8, 12, and 16 post-wounding, and biochemical markers were quantified. (**A**) Uronic acid content (µg/100 mg dry tissue), a marker of glycosaminoglycan synthesis. LT-treated groups, particularly the 10% LT group, showed significantly higher uronic acid levels compared with controls. (**B**) Hexosamine content (µg/100 mg dry tissue), reflecting proteoglycan and glycoprotein turnover. Both LT groups exhibited elevated hexosamine levels, peaking on Day 12. (**C**) Hydroxyproline content (µg/100 mg dry tissue), a surrogate marker of collagen deposition. LT-treated groups displayed markedly higher hydroxyproline levels, indicating enhanced collagen synthesis and remodeling. (**D**) Total protein content (µg/100 mg wet tissue), reflecting cellular proliferation and granulation tissue activity. Both 5% and 10% LT treatments significantly increased protein levels relative to control and SOB groups, with maximal levels observed on Day 12. Data are expressed as mean ± SEM (*n* = 6). Statistical analysis was performed using two-way ANOVA followed by Tukey’s multiple comparison post hoc test. * *p* < 0.05, ** *p* < 0.01, *** *p* < 0.001; ns, not significant.

Uronic acid levels, indicative of glycosaminoglycan synthesis, were significantly elevated in LT-treated groups compared with controls. The 10% LT group achieved the highest uronic acid concentration on Day 12 (6.91 ± 0.3 mg/g), exceeding levels observed in Silverex™-treated animals and maintaining significantly higher values than control and SOB groups throughout the study (*p* < 0.001) (Figure 5A). Similarly, hexosamine content, a marker of proteoglycan and glycoprotein biosynthesis, increased markedly in a time-dependent manner, peaking on Day 12 in the 10% LT group (5.83 ± 0.4 mg/g). While Silverex™ also promoted matrix formation, the 10% LT formulation produced a significantly greater elevation (*p* < 0.001), reflecting enhanced ECM remodeling and dermal regeneration (Figure 5B).

Hydroxyproline content, serving as a direct surrogate for collagen deposition, progressively increased across all treatment groups but was highest in the 10% LT group (8.29 ± 0.5 mg/g on Day 12), surpassing Silverex™, 5% LT, SOB, and control groups (*p* < 0.001) (Figure 5C). This finding highlights superior collagen biosynthesis and cross-linking, consistent with the higher tensile strength observed in LT-treated wounds. Total protein content, reflecting fibroblast proliferation and granulation tissue activity, followed a similar trend, with the 10% LT group reaching a peak of 12.7 ± 0.8 mg/g on Day 12, significantly higher than all other groups, including Silverex™ (*p* < 0.001) (Figure 5D).

Statistical analysis confirmed significant differences (*p* < 0.05) across both treatment groups and time points for all four ECM markers. Among the parameters, wound contraction exhibited the largest effect size (F = 42.26, R^2^ = 0.8879), followed by total protein (F = 23.47, R^2^ = 0.8149), hydroxyproline (F = 20.47, R^2^ = 0.7933), and hexosamine (F = 14.77, R^2^ = 0.7346). Although uronic acid showed a smaller F value (F = 3.315, *p* = 0.047), it still demonstrated a significant time-dependent trend (R^2^ = 0.3833) (Supplementary Table S3). Homogeneity of variance was confirmed for most parameters via Brown–Forsythe and Bartlett’s tests, except for uronic acid, which exhibited mild heteroscedasticity (Bartlett’s *p* = 0.039).

Post hoc Tukey’s multiple comparisons further revealed highly significant, time-dependent increases for all biochemical markers. Levels of uronic acid, hexosamine, hydroxyproline, and total protein were markedly elevated at Days 8, 12, and 16 relative to Day 4 (*p* < 0.001), demonstrating progressive ECM deposition and sustained tissue remodeling. Importantly, while wound contraction plateaued between Days 12 and 16, ECM-related biochemical markers continued to increase, emphasizing the 10% LT formulation’s ability to sustain molecular processes vital for long-term dermal repair.

### 2.7. Histopathological Evaluation of Wound Healing

**Figure 6:**
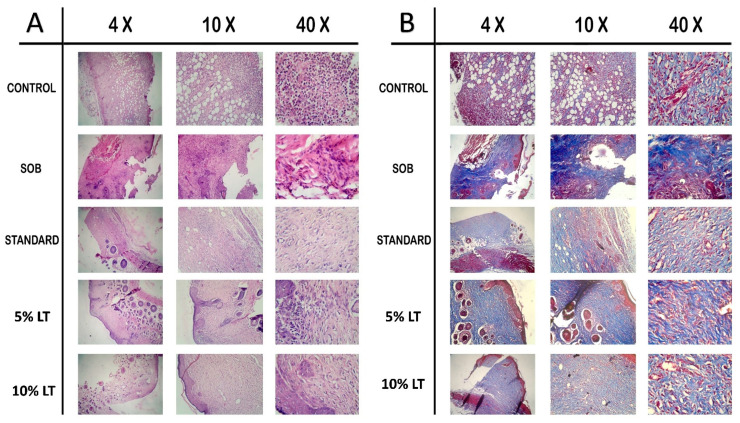
Histopathological analysis of wound tissues collected on Day 16 post-treatment in rats. Representative micrographs show hematoxylin and eosin (H&E) and Masson’s trichrome staining of wound tissues from the control, SOB (simple ointment base), standard (Silverex™), 5% LT, and 10% LT treatment 4× (scale bar = 500 µm), 10× (scale bar = 200 µm), and 40× (scale bar = 50 µm) magnifications. (**A**) H&E staining: The control and SOB groups displayed incomplete epithelialization, persistent inflammatory cell infiltration, and disorganized granulation tissue. The standard and LT-treated groups showed improved epithelial regeneration, reduced inflammation, and organized dermal architecture. The 10% LT group exhibited a fully developed stratified epithelium, minimal inflammatory infiltrates, and dense fibroblast proliferation, indicating advanced wound healing. (**B**) Masson’s trichrome staining: The control and SOB groups exhibited sparse, loosely arranged collagen fibers, whereas LT-treated groups, particularly the 10% LT group, showed dense, well-aligned collagen bundles with intense blue staining, reflecting enhanced extracellular matrix remodeling and collagen maturation.

Histological analysis on Day 16 post-treatment provided clear morphological evidence supporting the superior wound-healing potential of *Lobelia alsinoides* extract ointment compared to the reference standard, Silverex™. Evaluation using hematoxylin and eosin (H&E) and Masson’s trichrome staining (MTS) (Figure 6) revealed distinct differences across treatment groups in epithelial regeneration, inflammatory resolution, fibroblast proliferation, neovascularization, and collagen remodeling.

In the untreated control group, H&E-stained sections (Figure 6A) displayed incomplete re-epithelialization, persistent neutrophilic infiltration, hemorrhagic foci, and poorly organized granulation tissue, reflecting impaired repair processes. The SOB-treated wounds showed similar pathological features with disrupted epidermal layers, cellular degeneration, and residual inflammation, indicative of delayed healing.

By comparison, LT-treated wounds demonstrated progressive structural restoration, with the 10% LT formulation exhibiting the most advanced healing phenotype. The 10% LT-treated group showed a completely re-epithelialized epidermis with a continuous keratinized layer, well-organized dermal fibroblast alignment, and minimal inflammatory infiltration. Neovascularization was pronounced, evidenced by dense capillary networks and active angiogenic foci, signifying accelerated tissue regeneration. Compared to Silverex™, the 10% LT group exhibited a more compact, mature granulation tissue architecture, reduced inflammatory cell density, and superior fibroblast organization, suggesting faster transition from the proliferative to the remodeling phase.

Masson’s trichrome staining (MTS) (Figure 6B) further corroborated these findings. While the control and SOB groups exhibited sparse, loosely arranged collagen bundles with weak blue staining, Silverex™-treated sections displayed moderately organized collagen fibers with partial maturation. In contrast, the 10% LT-treated tissues showed intensely stained, densely packed, and uniformly aligned collagen fibers throughout the dermal layer, reflecting enhanced extracellular matrix deposition and advanced structural remodeling. The 5% LT formulation also demonstrated substantial improvement compared with control and SOB groups, though the 10% LT ointment achieved superior collagen maturation relative even to Silverex™, underscoring its potential as a high-efficacy phytotherapeutic for wound repair.

### 2.8. Systemic Safety Assessment of L. alsinoides Lam Ethanolic Extract Formulations

Systemic safety evaluation of *L. alsinoides* ethanolic extract ointments revealed that topical application of both 5% and 10% LT formulations was not only non-toxic but also demonstrated hepatoprotective and renoprotective trends superior to the reference standard, Silverex™. Key biochemical markers of hepatic and renal function were assessed on Day 16 post-treatment and compared across control, SOB, Silverex™, and LT-treated groups (Supplementary Table S4).

No evidence of systemic toxicity was observed in any LT-treated animals. Instead, the 10% LT ointment group exhibited significantly reduced levels of hepatic and renal stress markers, indicating enhanced systemic safety. Total bilirubin, a sensitive indicator of hepatic dysfunction, was markedly decreased in the 10% LT group (0.35 ± 0.13 mg/dL) compared with both the untreated control (0.81 ± 0.13 mg/dL) and Silverex™ group (0.47 ± 0.09 mg/dL). This decline suggests a lower hepatic load and possible hepatoprotective activity of LT relative to the silver-based reference. Likewise, total serum protein levels normalized toward physiological ranges in LT-treated rats (4.9 ± 0.22 g/dL for 10% LT), supporting improved protein metabolism and systemic homeostasis during wound resolution.

Liver enzyme assays further highlighted the superior safety profile of LT ointment. Serum Aspartate Aminotransferase (AST/SGOT) and Alanine Aminotransferase (ALT/SGPT) levels were markedly reduced in LT-treated animals, indicating minimal hepatic stress. The 10% LT group recorded AST and ALT values of 32.18 ± 2.24 U/L and 21.91 ± 0.31 U/L, respectively, compared to 58.12 ± 4.12 U/L and 47.30 ± 2.43 U/L in controls, and slightly lower than the corresponding values observed with Silverex™ treatment. These findings imply that LT not only matches but may exceed Silverex™ in preserving hepatic integrity following topical application.

Renal function parameters corroborated these hepatoprotective effects. Urea and creatinine levels were substantially lower in LT-treated groups, with the 10% LT group exhibiting the lowest mean concentrations (22.27 ± 0.57 mg/dL and 0.52 ± 0.15 mg/dL, respectively). These values were comparable to or slightly better than those in the Silverex™ group and markedly improved relative to SOB and control groups, confirming the absence of nephrotoxicity and demonstrating the formulation’s systemic safety.

## 3. Discussion

The present study builds upon and advances earlier investigations on *Lobelia* species, including our previous work demonstrating burn wound healing by *L. alsinoides* ethanolic extract [26], the nanoscale enhancement of *Lobelia trigona*-based formulations [28], and phytochemical and cytotoxic evaluations [23,27]. While these studies established the therapeutic potential of *Lobelia* extracts in partial-thickness or thermal wound models, they did not address the mechanistic basis of healing in deep or full-thickness wounds. The current investigation bridges this gap through a comprehensive evaluation encompassing phytochemical characterization, in vitro cytocompatibility, antimicrobial profiling, and in vivo full-thickness excision and incision models, culminating in a demonstration of LT’s superior wound-healing efficacy compared to the established silver-based formulation, Silverex™.

The *L. alsinoides* ethanolic extract is notably enriched with phenolics, flavonoids, terpenoids, tannins, and alkaloids, all of which play central roles in wound repair through synergistic antioxidant, anti-inflammatory, and antimicrobial actions [29,30]. The abundance of polyphenols contributes directly to enhanced granulation tissue formation, angiogenesis, and accelerated wound closure, while flavonoids and terpenoids act as potent ROS scavengers and modulators of inflammatory signaling pathways. Compared to Silverex™, which primarily acts through topical antimicrobial action via ionic silver release [31], LT provides a multifactorial therapeutic effect combining antimicrobial potency with tissue-protective and regenerative mechanisms.

The broad antibacterial spectrum of *L. alsinoides* extract aligns with antimicrobial activities reported in related *Lobelia* species and other medicinal plants [19,28]. Essential oils from *Lobelia pyramidalis* demonstrated moderate activity against *S. aureus* and *Trichophyton* species, mainly attributed to terpenoid constituents such as perilla ketone, known for disrupting microbial membranes [32]. By comparison, the ethanolic extract of *L. alsinoides* showed stronger potency with lower MIC values. The extract inhibited both Gram-positive and Gram-negative bacteria, including resistant strains such as MRSA and *A. baumannii*, highlighting its clinical relevance. Similar broad-spectrum efficacy has been reported for plant extracts rich in phenolics, flavonoids, and terpenoids, which act by disrupting membranes, interfering with quorum sensing, and inhibiting biofilm formation [33]. Its fungistatic activity against *Candida* species and *Trichophyton rubrum* is consistent with the known antifungal mechanisms of polyphenols and terpenoids, which disrupt fungal cell walls and interfere with metabolism [34]. The *L. alsinoides* extract exhibited MIC values ranging from 9.67 ± 0.58 to 17.67 ± 0.58 µg/mL and MBC/MFC values from 24.67 ± 0.58 to 27.67 ± 0.58 µg/mL, demonstrating potent bactericidal and fungicidal properties. The pronounced activity against MRSA, *A. baumannii*, and *A. fumigatus* highlights the extract’s therapeutic potential as a broad-spectrum antimicrobial agent. The results presented in Figure S3 further illustrate this trend, with heat maps showing lower MIC, MBC, and MFC values corresponding to stronger antimicrobial potency across bacterial and fungal species. The MIC values fall within therapeutic ranges reported for natural antifungal agents, further supporting *L. alsinoides* as a promising topical antimicrobial alternative or adjunct for managing infected wounds. These results are consistent with the identified phenolic, flavonoid, and terpenoid constituents, which are known to contribute to microbial membrane disruption and oxidative stress-mediated inhibition [35].

The potent antioxidant activity observed in *L. alsinoides* aligns with findings from related *Lobelia* species and other medicinal plants, primarily attributed to their abundant phenolic and flavonoid content [24,28,36,37,38]. Studies on *Lobelia chinensis* and *Lobelia nicotianifolia* report significant in vitro antioxidant effects, including strong DPPH and ABTS radical scavenging, linked to polyphenols and flavonoids that protect against lipid peroxidation and oxidative damage [36,39,40,41]. These antioxidants also contribute to hepatoprotective effects by enhancing endogenous defense systems, such as superoxide dismutase (SOD) activity, providing systemic benefits beyond free radical neutralization [42]. The IC_50_ values for DPPH and superoxide radical scavenging in LT are comparable to those of well-known antioxidants, indicating effective hydrogen donation and electron transfer capabilities crucial for interrupting radical chain reactions during oxidative stress [43]. This highlights the importance of robust antioxidant defense as a common mechanism supporting medicinal plant-mediated wound repair.

The consistent R^2^ values (0.9694–0.9884) across DPPH, hydroxyl, and superoxide radical models validate the reproducibility of the fitted nonlinear regression curves and confirm the robustness of the experimental data. Among the tested ROS, superoxide radicals were most efficiently neutralized (IC_50_ = 9.40 µg/mL, 95% CI: 6.36–13.15 µg/mL), reflecting the extract’s superior electron-donating potential. In comparison, DPPH and hydroxyl radicals exhibited IC_50_ values of 15.65 µg/mL (95% CI: 8.97–25.96 µg/mL) and 84.78 µg/mL (95% CI: 46.58–196.3 µg/mL), respectively. The moderate hydroxyl radical scavenging capacity reflects the higher reactivity of hydroxyl radicals and the distinct antioxidant mechanisms required for their neutralization. The extract’s low IC_50_ for superoxide radicals highlights its strong reducing power and the presence of potent electron-donating phytoconstituents, likely phenolics and flavonoids, capable of donating hydrogen atoms or electrons to stabilize free radicals. Although less pronounced for hydroxyl radicals, the extract’s inhibitory effect still contributes meaningfully to the overall antioxidant defense network by intercepting hydroxyl-induced oxidative chain reactions.

The *L. alsinoides* ethanolic extract exhibited favorable cytocompatibility with HaCaT keratinocytes, maintaining cell viability above 90% at concentrations below 20 µg/mL, which falls within the accepted safety threshold for topical formulations. This confirms a broad safety margin and supports the suitability of *L. alsinoides* extract for further wound-healing and skin regeneration studies. The brine shrimp lethality assay further confirmed the extract’s low systemic toxicity, reinforcing its safety profile. The extract significantly enhanced keratinocyte migration in scratch assays, a crucial process in wound re-epithelialization that depends on keratinocyte proliferation and motility. This effect aligns with reports that phenolic-, flavonoid-, and terpenoid-rich plant extracts stimulate HaCaT migration and facilitate faster wound closure by modulating growth factors and cytokines [44,45]. The dose-dependent response suggests potential for optimized therapeutic formulations. Similar cytocompatibility and promotion of keratinocyte migration have been documented for plants such as *Centella asiatica* and *Aloe vera*, highlighting the advantage of phytotherapeutics over certain antiseptics that impair keratinocyte viability [46,47].

In vivo safety data confirmed the absence of systemic toxicity associated with *L. alsinoides* ethanolic extract, consistent with plant-based topical therapeutics rich in phenolics and flavonoids that exert antioxidative and anti-inflammatory effects while preserving healthy tissue integrity [48]. The enhanced wound-healing efficacy observed—manifested by accelerated closure, faster epithelialization, and greater tensile strength—correlates with earlier findings from *L. alsinoides* in burn models [26], yet surpasses those outcomes in both magnitude and reproducibility. Topical application of the 10% LT ointment markedly enhanced collagen synthesis, fibroblast proliferation, and overall structural integrity of the healed tissue, exceeding the performance of the Silverex™ and reaffirming the traditional therapeutic value of *Lobelia* species in regenerative medicine.

Elevated levels of uronic acid, hexosamine, and hydroxyproline in LT-treated tissues further validate the extract’s role in promoting ECM synthesis and remodeling, fundamental processes in dermal repair. The pronounced increases in uronic acid and hexosamine indicate robust biosynthesis of glycosaminoglycans and proteoglycans, which maintain tissue hydration, enhance cell adhesion, and regulate fibroblast migration [49]. Concurrently, the significant elevation of hydroxyproline, a direct marker of collagen content, demonstrates active fibroblast proliferation and collagen deposition, essential for dermal strength and elasticity [50]. Compared to Silverex™, LT-treated wounds exhibited consistently higher hydroxyproline and total protein levels, signifying more advanced matrix maturation and structural restoration.

Histological analyses corroborated these biochemical results, showing well-organized collagen bundles, mature fibroblast populations, and advanced tissue remodeling in LT-treated wounds. The 10% LT formulation, in particular, induced dense, uniformly aligned collagen fibers, fully stratified epidermal architecture, and extensive neovascularization, hallmarks of complete dermal restoration. These histopathological features were more pronounced than those observed in Silverex™-treated tissues, which exhibited comparatively lower collagen density and less compact matrix organization. The reduced inflammatory infiltrates in LT-treated sections highlight its combined anti-inflammatory, antioxidant, and antimicrobial mechanisms, which together create an optimal microenvironment for rapid and scar-minimized tissue regeneration [51,52,53].

Systemic biochemical parameters further underscored LT’s superiority in safety. The 10% LT-treated group showed significant reductions in SGOT and SGPT levels, indicative of hepatoprotection consistent with the bioactivity of phenolics, flavonoids, and alkaloids reported in *Lobelia* species [24,54]. Similarly, reduced serum urea and creatinine levels reflected preserved renal function and absence of nephrotoxicity. Notably, these hepatoprotective and renoprotective effects were comparable to, or slightly superior to, those seen in Silverex™-treated animals, establishing LT as not only efficacious but also exceptionally biocompatible for repeated topical use.

This study has certain limitations. Although robust histological and biochemical evidence confirmed enhanced ECM remodeling and angiogenesis, a formal correlation analysis between ECM biomarkers and tensile strength was not performed because these parameters were obtained from distinct wound models and non-paired samples; however, both endpoints showed concordant group-level trends, suggesting a functional relationship between collagen deposition and biomechanical recovery. Molecular delineation of the regulatory signaling cascades, particularly TGF-β/Smad, VEGF, NF-κB, and Nrf2, was not included. Nonetheless, the observed biochemical markers (hydroxyproline, uronic acid, and hexosamine) and histological features (fibroblast proliferation, neovascularization, and reduced inflammatory infiltrates) are consistent with reported mechanisms through which polyphenol- and flavonoid-rich phytotherapeutics activate TGF-β/Smad-dependent fibroblast differentiation, stimulate VEGF-mediated angiogenesis, suppress NF-κB-driven inflammation, and enhance Nrf2-regulated antioxidant responses [15,16,29,30,45,55].

Future studies should therefore integrate molecular analyses (qRT-PCR, ELISA, and Western blotting) targeting TGF-β1/Smad2/3, VEGF-A, COL1A1/COL3A1, NF-κB p65, and Nrf2/HO-1 to validate these pathways and elucidate the mechanistic basis of *L. alsinoides*-mediated wound repair. Moreover, evaluation in clinically relevant chronic and infected wound models, comprehensive antimicrobial assays (biofilm disruption, antibiotic synergy, and time-kill kinetics), bioassay-guided fractionation of active phytoconstituents, and long-term safety and pharmacokinetic studies will further advance its translational potential. A focused translational roadmap incorporating mechanistic investigations, chronic wound models, and early-phase clinical trials will facilitate the progression of LT ointment toward evidence-based wound care applications.

Given the multi-constituent nature of botanical extracts, the observed wound-healing effects are ascribed to the standardized extract as an integrated phytochemical system; definitive attribution to specific constituents awaits bioassay-guided fractionation and targeted quantitative analyses. Future studies will perform bioassay-guided fractionation with LC-HRMS/MS-guided dereplication and 2D-NMR structural confirmation, dereplicate active fractions (LC-HRMS/MS molecular networking) and isolate leads, confirm structures by 1D/2D-NMR (^1^H, ^13^C, HSQC, HMBC, COSY) and HR-MS/MS, and verify with authentic standards when available. Confirmed actives will be quantified in the bulk extract and ointment by targeted LC-MS/MS (MRM/SRM) and/or HPLC-UV using calibration curves, LOD/LOQ, and recovery validation. Reconstitution/add-back experiments will determine whether identified constituents (alone or in combination) recapitulate the crude-extract activity and assess potential synergy using isobologram/FIC index and Bliss/Loewe analyses. These investigations will establish bioactive constituents and quantitative dose-effect relationships underlying the wound-healing efficacy of *L. alsinoides* Lam.

## 4. Materials and Methods

### 4.1. Extraction and Quantitative Phytochemical Analysis

Whole plants of *Lobelia alsinoides* Lam. (LT) were collected from the Palakkad district of Kerala, India, in October 2014 (latitude 10°48′24.42″ N; longitude 76°11′47.35″ E). Botanical authentication was performed using established taxonomic criteria and confirmed at the Botanical Survey of India (BSI), TNAU campus, Coimbatore, India. The authenticated specimen was deposited under voucher number BSI/SRC/5/23/2014-15/Tech/1143 [27]. Fresh, disease-free plants were thoroughly washed, shade-dried at room temperature, and ground to a coarse powder. Soxhlet extraction was performed with 95% ethanol at 60–65 °C for 8 h, using a 1:10 (*w*/*v*) plant-to-solvent ratio. Ethanol was removed by rotary evaporation under reduced pressure, and the crude extract was stored at 4 °C until use. The percentage yield of the ethanolic extract was determined according to established procedures [27].

Quantitative phytochemical profiling was performed using standard spectrophotometric methods. Concentrations were standardized and expressed as equivalents of reference standards: total phenolic content as mg gallic acid equivalents (GAE)/g extract [56]; total flavonoid content as mg rutin equivalents (RE)/g [57]; total tannin content as mg GAE/g [58]; total alkaloid content as mg atropine equivalents (AE)/g [59]; total saponin content as mg ginsenoside equivalents (GSE)/g [60]; and total terpenoid content as mg ursolic acid equivalent (UAE)/g [61]. All analyses were carried out in triplicate (*n* = 3).

The phytochemical profile and spectral fingerprint of the *L. alsinoides* ethanolic extract used in this study were previously characterized by our group using LC-QTOF-MS, FTIR, and ^1^H/^13^C NMR analyses [23] which provided the reference fingerprint for confirming extract identity and ensuring batch-to-batch reproducibility in the present work. No new compound identifications are reported herein.

### 4.2. Antibacterial and Antifungal Activity

For all antimicrobial assays, LT extract was dissolved in dimethyl sulfoxide (DMSO) (Cat#D8418, Sigma-Aldrich, Bengaluru, India); the final DMSO concentration was ≤0.5% *v*/*v* (never exceeding 1% *v*/*v*). A vehicle-only control (DMSO at the corresponding final % *v*/*v*) and a medium-only negative control were included on every plate.

Antimicrobial screening [62] was performed against 13 bacterial strains, including six Gram-positive bacteria: *Bacillus subtilis* (MTCC 441), *Staphylococcus epidermidis* (MTCC 10623), *Streptococcus pneumoniae* (MTCC 1936), *Lactobacillus acidophilus* (MTCC 10307), *Staphylococcus aureus* (MTCC 3160), methicillin-resistant *Staphylococcus aureus* (MRSA, MTCC 1430); and seven Gram-negative bacteria: *Klebsiella pneumoniae* (MTCC 3384), *Acinetobacter baumannii*, *Pseudomonas aeruginosa* (MTCC 4676), *Escherichia coli* (MTCC 443), *Salmonella paratyphi* (MTCC 3220), and *Shigella sonnei* (MTCC 2957). Due to diffusion limitations for filamentous fungi, antifungal susceptibility was assessed exclusively by broth microdilution. Fungal strains included *Aspergillus flavus* (MTCC 3382), *Aspergillus fumigatus* (MTCC 343), *Aspergillus niger* (MTCC 281), *Candida albicans* (MTCC 183), *Candida tropicalis* (MTCC 230), and *Trichophyton rubrum* (MTCC 296). All strains were from the Institute of Microbial Technology (IMTECH), CSIR, Chandigarh, India.

Bacteria were maintained on nutrient agar (NA; Cat#M001, HiMedia, Thane, India) and fungi on potato dextrose agar (PDA; Cat#M096A, HiMedia, Thane, India), stored at 4 °C. Agar well diffusion assays were performed at extract concentrations of 10–100 µg/mL. Gentamicin (10 µg; Cat#TC026, HiMedia, Thane, India) was the positive control for bacteria, fusidic acid (Cat#SD171, HiMedia, Thane, India) specifically for MRSA, and ketoconazole (10 µg; Cat#SD224, HiMedia, Thane, India) for fungi in complementary assays.

Minimum inhibitory concentrations (MICs), minimum bactericidal concentrations (MBCs), and minimum fungicidal concentrations (MFCs) were determined by broth microdilution [63]. For bacteria, MIC/MBC followed CLSI guidelines with two-fold serial dilutions (5–100 µg/mL). For yeasts and filamentous fungi, MIC/MFC determinations adhered to CLSI M27 and M38, respectively, including standardized inoculum preparation, incubation times (24–48 h for yeasts; 48–72 h for molds), and endpoints. MFCs were confirmed by subculturing from non-turbid wells onto fresh agar. All experiments were performed in triplicate (*n* = 3), and zones of inhibition and MIC values are reported as mean ± SEM.

### 4.3. Antioxidant Activity

Multiple in vitro assays were conducted to determine the antioxidant potential of the LT ethanolic extract. The DPPH radical scavenging assay was carried out by incubating 1.0 mL of 0.3 mM DPPH (MB263) (Cat#MB263, HiMedia, Thane, India India) solution with 1.0 mL of LT extract at various concentrations and 1.0 mL of methanol in the dark for 10 min, followed by absorbance measurement at 517 nm. Ascorbic acid (Cat#TC094, HiMedia, Thane, India, India) was used as the positive control [64]. The hydroxyl radical scavenging activity was assessed using the Fenton reaction, in which a mixture containing 2-deoxy-D-ribose (Cat#TC114, HiMedia, Thane, India), Ethylenediaminetetraacetic acid (EDTA) (Cat#GRM1195, HiMedia, Thane, India), ferric chloride (FeCl_3_) (Cat#GRM1178, HiMedia, Thane, India), hydrogen peroxide (H_2_O_2_) (Cat#PCT1511, HiMedia, Thane, India India), ascorbic acid, and LT extract was incubated, followed by reaction with thiobarbituric acid (TBA) (Cat#GRM1594, HiMedia, Thane, India) and trichloroacetic acid (TCA) (Cat#GRM7570, HiMedia, Thane, India). Absorbance was recorded at 532 nm, with gallic acid as a standard [65]. For the superoxide anion radical scavenging assay, a reaction mixture containing Nitroblue Tetrazolium (NBT) (Cat#MB107, HiMedia, Thane, India), Tris-HCl buffer (pH 8) (Cat#TC073, HiMedia, Thane, India), Nicotinamide Adenine Dinucleotide (NADH) (Cat#MB270, HiMedia, Thane, India), phenazine methosulfate (PMS) (Cat#TC026, HiMedia, Thane, India), and LT extract was prepared and incubated at 25 °C for 5 min, with absorbance measured at 560 nm. Gallic acid (Cat#PCT1546, HiMedia, Thane, India) was used as the reference standard [66]. The reducing power of LT was measured by incubating LT extract with phosphate buffer and potassium ferricyanide (Cat#GRM1034, HiMedia, Thane, India) at 50 °C, followed by the addition of TCA, centrifugation, and mixing with distilled water. The absorbance was recorded at 700 nm with ascorbic acid as the reference [67].

### 4.4. Brine Shrimp Lethal Toxicity Assay

Preliminary toxicity was assessed using the brine shrimp lethality assay. Second-instar *Artemia salina* nauplii (~2 days old) were exposed to graded concentrations of LT extract prepared in DMSO-saline and distributed into 96-well plates. Mortality was recorded at 6, 12, and 24 h post-exposure, and the lethal concentration 50% (LC_50_) values were calculated [68]. Each concentration was tested in six replicates (*n* = 6).

### 4.5. In Vitro Cytotoxicity Assay

Cytotoxic potential was evaluated by the 3-(4,5-dimethylthiazol-2-yl)-2,5-diphenyl-2H-tetrazolium bromide (MTT) assay on HaCaT human keratinocyte cells. Cells (1 × 10^4^/well) were seeded in 96-well plates and allowed to adhere overnight, then treated with LT extract (6.25–100 μg/mL) and incubated for 24 h under standard culture conditions. Following treatment, MTT reagent (Cat#RM1131, HiMedia, Thane, India) was added to each well and incubated to allow for formazan formation. Absorbance was measured at 540 nm, and results were expressed as percentage viability relative to untreated controls [69]. All experiments were performed in triplicate (*n* = 3). Antioxidant assay outcomes are reported as IC_50_ values in µg/mL to match crude-extract dosing units.

### 4.6. In Vitro Scratch Assay

In vitro wound-healing potential was assessed using a scratch assay in HaCaT cells (National Centre for Cell Science (NCCS), Pune, India; Cell Repository Code: NCCS-HaCaT; passage numbers: 5 to 10). Cells were cultured in Dulbecco’s Modified Eagle Medium (DMEM) (Cat#AL269A, HiMedia, Thane, India) supplemented with 10% fetal bovine serum (FBS) (Cat#RM9955, HiMedia, Thane, India), 100 U/mL penicillin (Cat#TC187, HiMedia, Maharashtra, India), and 100 µg/mL streptomycin (Cat#TC035, HiMedia, Thane, India) at 37 °C in a humidified atmosphere containing 5% CO_2_. For the assay, cells were seeded in 6-well plates at a density of 2 × 10^5^ cells/well in DMEM supplemented with 1% FBS to minimize proliferation and grown to confluence overnight.

A linear scratch was made with a sterile 200 µL pipette tip, and debris was removed with phosphate-buffered saline (PBS) washes. Monolayers were treated with LT (10, 50, or 100 µg/mL) in medium containing 0.1% DMSO. Controls included untreated cells and vehicle control (0.1% DMSO). Plates were incubated for 24 h at 37 °C, 5% CO_2_ [70]. Phase-contrast images were captured at 0 and 24 h (20×). Percent wound closure was quantified by scratch width/area reduction using Image J software (Version 1.51) (National Institutes of Health (NIH), Bethesda, USA). Each condition was tested in triplicate (*n* = 3).

### 4.7. Formulation of LT Ointment

The topical LT ointment used a standard emulsifying base: wool fat (5%), hard paraffin (5%), cetostearyl alcohol (5%), and white soft paraffin (85%). The base was prepared by the fusion method: hard paraffin and cetostearyl alcohol were melted at 70 °C, followed by incorporation of wool fat and white soft paraffin with gentle trituration. The ethanolic extract of *L. alsinoides* was incorporated at 5% and 10% (*w*/*w*) under constant stirring at 40–45 °C to obtain a homogeneous mixture. Formulations were cooled, transferred into sterile airtight containers, and stored at ambient conditions.

To ensure consistency, stability, and suitability for topical application, key physicochemical parameters were systematically evaluated. Organoleptic characteristics (color, odor, texture, and homogeneity), pH (1% aqueous dispersion measured with a calibrated digital pH meter), spreadability (parallel-plate method), and viscosity (Brookfield viscometer, spindle no. 64 at 25 °C) were assessed. Homogeneity was confirmed through visual and microscopic inspection to ensure uniform extract distribution and absence of phase separation. Stability was assessed at 25 °C and 37 °C for up to 12 months. No microbial contamination, discoloration, odor change, or texture alteration was observed. Formulations maintained skin-compatible pH (5.8–6.4) and spreadable consistency.

### 4.8. Animal Study Design and Wound Models

Adult male Wistar rats (8–10 weeks, 180–200 g) were randomly assigned to five groups: (1) untreated control; (2) vehicle control (simple ointment base, SOB); (3) standard control (Silverex™, 1% *w*/*w* silver sulfadiazine, 0.2% *w*/*w* chlorhexidine; Sun Pharmaceutical Industries Ltd., India); (4) 5% LT ointment; and (5) 10% LT ointment. Each animal served as an independent experimental unit. The sample size (*n* = 6 per group per experiment) followed validated full-thickness wound-healing studies reporting comparable variability; no a priori power calculation was performed.

Healthy, immunocompetent rats were obtained from the CPCSEA-registered Central Animal House Facility (Kerala Veterinary and Animal Sciences University, Mannuthy, Kerala, India) and acclimatized for seven days (polypropylene cages, 12 h light/dark, standard pellet diet, water ad libitum; environmental enrichment provided). All procedures were approved by the Institutional Animal Ethics Committee (IAEC/KU/BT/14/23) and adhered to CPCSEA guidelines. Prior to efficacy testing, acute dermal toxicity was assessed with a topical dose of 2000 mg/kg LT extract.

Randomization was conducted using a random-sequence number managed by an investigator not involved in data collection. Cage positions were rotated weekly to minimize environmental confounders. Treatment codes were concealed in labeled containers. The dosing investigator was necessarily aware of assignments to ensure accuracy, whereas wound photography, planimetry, tensile testing, biochemical assays, and histological evaluations were performed by independent observers blinded to group allocation. All data files were coded and decoded only after completion of statistical analysis.

Two wound models were used. Incision model: a 1.5 cm linear paravertebral incision was made under anesthesia and sutured with nylon; treatments were applied daily for 10 days; tensile strength was measured on Day 10. Excision model: a full-thickness circular wound (~100 mm^2^) was created on the dorsal thoracic region; treatments were applied daily until complete epithelialization or Day 16. Wound contraction was assessed on Days 0, 4, 8, 12, and 16.

To ensure reproducibility, wound areas were evaluated by two blinded observers. Standardized images were captured at each time point to Day 16, and contraction was quantified using Image J software (Version 1.51) (National Institutes of Health (NIH), Bethesda, USA); direct measurements with a transparent millimeter-scale grid were also obtained. All measurements were performed in triplicate and averaged per animal/time point.

### 4.9. Tensile Strength Measurement

Mechanical integrity of healed skin (incision model) was assessed on postoperative Day 10. Full-thickness skin strips from the wound site were subjected to uniaxial tension using an Instron-type 6021 tensiometer (1 kN piezoresistive load cell; 5000 Hz acquisition). Each specimen was secured using anti-slip clamps to prevent slippage, and the crosshead speed was maintained at 100 mm/min. The breaking strength, the force required to disrupt the healed tissue, was recorded in Newtons and converted to grams for intergroup comparison. Measurements were expressed as mean ± SEM (*n* = 6/group/experiment).

### 4.10. Wound Contraction and Epithelialization Period

In the excision model, wound area was measured on alternate days using a transparent millimeter-scale grid. Wound contraction (%) was calculated as: Wound contraction = ((Initial wound area 2212 current wound area)/Initial wound area) × 100. This metric reflects the rate at which the wound surface reduced over time. The epithelialization period was recorded as the number of days required for complete scab (eschar) detachment without residual raw tissue.

### 4.11. Biochemical and Biotoxicity Assays

Granulation tissues were excised on Days 4, 8, 12, and 16 to evaluate ECM-related biochemical markers. Uronic acid, hexosamine, hydroxyproline (a surrogate of collagen content), and total protein were quantified using standard spectrophotometric methods. Results are expressed as mg/g wet tissue and analyzed in triplicate (*n* = 3/time point). Systemic safety of LT formulations was assessed on Day 16 from blood serum. Liver function was evaluated by SGOT/AST, SGPT/ALT, alkaline phosphatase (ALP), total bilirubin, and total serum protein; renal function by urea and creatinine. All assays were performed in triplicate.

### 4.12. Histopathological Examination

On Day 16, excision-model skin tissues were collected for histology. Specimens were fixed in 10% neutral-buffered formalin for 24 h, processed, and paraffin-embedded. Sections (5 µm) were prepared on glass slides. Hematoxylin and eosin (H&E) was used to assess tissue architecture, epithelial regeneration, inflammatory cell infiltration, neovascularization, and fibroblast activity. Masson’s trichrome staining (MTS) evaluated collagen fiber organization and density. Slides were examined by light microscopy, and representative images were documented across groups.

### 4.13. Statistical Analysis

All experimental data were expressed as mean ± standard error of the mean (SEM) from at least three independent determinations (*n* = 3/group/experiment for in vitro assays and *n* = 6/group/experiment for in vivo experiments). Statistical analyses and graphical visualizations, including dose–response curves, bar plots, and color-coded heat maps, were generated using GraphPad Prism version 10.0 (GraphPad Software, San Diego, CA, USA).

Dose–response relationships for antioxidant (DPPH, hydroxyl, and superoxide radical scavenging) and cytotoxicity assays were fitted using a three-parameter nonlinear regression model ([Inhibitor] vs. Response) to calculate IC_50_ values, 95% confidence intervals (CI), and correlation coefficients (R^2^). Quantitative phytochemical data were analyzed using an unpaired *t*-test.

Comparisons among multiple groups for antimicrobial, biochemical, and wound healing parameters were performed using one-way or two-way analysis of variance (ANOVA) followed by Tukey’s multiple comparison post hoc test to identify intergroup differences. For comparisons between MIC, MBC, and MFC values across microbial strains, a two-way ANOVA with Bonferroni’s multiple comparison test was applied to determine concentration-dependent and organism-specific effects.

Normality and homogeneity of variances were verified using Bartlett’s and Brown-Forsythe tests prior to ANOVA. These assumptions were satisfied, and significant group differences were observed for all major wound-healing endpoints (*p* < 0.001), confirming statistical adequacy of the chosen sample size (Table S3).

Differences were considered statistically significant when *p* < 0.05. Significance levels were denoted as: * *p* < 0.05, ** *p* < 0.01, *** *p* < 0.001, **** *p* < 0.0001.

## 5. Conclusions

This study provides the first comprehensive pharmacological and safety evaluation of *L. alsinoides* Lam. ethanolic extract formulated as a topical ointment. Through integrated phytochemical, antimicrobial, antioxidant, cytocompatibility, and in vivo analyses, the 10% LT formulation demonstrated faster wound contraction, enhanced epithelialization, superior collagen remodeling, and greater systemic safety compared with the silver-based standard Silverex™. These findings establish LT as not only an effective but also a safer and more sustainable alternative to conventional silver therapies. Its multifaceted wound-healing mechanisms, encompassing antimicrobial, antioxidant, anti-inflammatory, and regenerative actions, offer a distinct therapeutic advantage over monotherapeutic silver formulations. With further mechanistic validation and clinical translation, LT holds strong potential as an evidence-based, affordable phytotherapeutic for integration into modern wound care practice.

## Figures and Tables

**Table 1:** Antibacterial and antifungal activity of *Lobelia alsinoides* Lam. ethanolic extract against selected microbial pathogens.

	Microbial Pathogens	Zone of Inhibition			
		Standard	25 µg (LT)	50 µg (LT)	100 µg (LT)
**Gram-Positive Bacteria**	*Bacillus subtilis*	20.33 ± 0.20	-	-	17.67 ± 1.92
	*Staphylococcus epidermidis*	19.66 ± 0.48	-	15.66 ± 1.92	18.00 ± 1.92
	*Streptococcus pneumoniae*	19.66 ± 0.88	-	-	17.67 ± 1.83
	*Lactobacillus acidophilus*	18.66 ± 0.33	-	13.00 ± 1.22	16.67 ± 1.88
	*Staphylococcus aureus*	19.66 ± 0.82	-	15.33 ± 1.28	17.67 ± 1.89
	MRSA	20.86 ± 0.63	11.33 ± 1.45	15.66 ± 1.39	18.33 ± 1.38
**Gram-Negative Bacteria**	*Acinetobacter baumannii*	19.56 ± 0.99	12.66 ± 1.22	15.33 ± 1.28	17.67 ± 1.55
	*Klebsiella pneumoniae*	20.66 ± 0.23	-	14.33 ± 1.89	19.00 ± 1.64
	*Escherichia coli*	20.96 ± 0.78	-	12.33 ± 1.28	17.33 ± 1.44
	*Pseudomonas aeruginosa*	21.00 ± 0.55	-	15.67 ± 1.93	17.66 ± 1.78
	*Salmonella paratyphi*	19.66 ± 0.89	-	13.66 ± 1.82	18.67 ± 1.72
	*Serratia marcescens*	19.33 ± 0.22	-	12.33 ± 1.44	16.67 ± 1.87
	*Shigella sonnei*	19.00 ± 0.19	-	13.66 ± 1.89	17.33 ± 1.37
**Fungi**	*Aspergillus flavus*	20.11 ± 0.39	07.10 ± 1.12	12.18 ± 1.75	17.58 ± 1.10
	*Aspergillus fumigatus*	20.48 ± 0.67	12.09 ± 1.85	15.18 ± 1.13	19.11 ± 1.78
	*Aspergillus niger*	20.33 ± 0.29	08.46 ± 1.82	13.26 ± 1.92	17.33 ± 1.77
	*Candida albicans*	20.86 ± 0.22	09.66 ± 1.65	13.00 ± 1.33	18.66 ± 1.78
	*Candida tropicalis*	19.34 ± 0.13	12.13 ± 1.11	16.54 ± 1.35	18.10 ± 1.51
	*Trichophyton rubrum*	20.11 ± 0.31	-	14.82 ± 1.12	18.65 ± 1.13

Note: Gentamicin was used as the standard for Gram-positive and Gram-negative bacteria, fusidic acid for methicillin-resistant Staphylococcus aureus (MRSA), and ketoconazole for fungal strains. The minimum inhibitory concentration (MIC) represents the lowest concentration that inhibits visible microbial growth. Values are expressed as the diameter of the inhibition zone (mm) or concentration (µg/mL), presented as mean ± SEM (*n* = 3). (-) indicates no detectable activity at the tested concentration.

## Data Availability

Data is contained within the article and Supplementary Materials.

## Supplementary Materials

The following supporting information can be downloaded at: https://www.mdpi.com/article/10.3390/ijms262110663/s1.

## Abbreviations

The following abbreviations are used in this manuscript:

AE	Atropine equivalents
ALP	Alkaline phosphatase
AMR	Antimicrobial resistance
ANOVA	Analysis of Variance
ALT (SGPT)	Alanine Aminotransferase/Serum Glutamic-Pyruvic Transaminase
AST (SGOT)	Aspartate Aminotransferase/Serum Glutamic-Oxaloacetic Transaminase
BSI	Botanical Survey of India
CI	Confidence Interval
CLSI	Clinical and Laboratory Standards Institute
COL1A1	Collagen Type I Alpha 1 Chain
COL3A1	Collagen Type III Alpha 1 Chain
CO_2_	Carbon Dioxide
CSIR	Council of Scientific & Industrial Research (India)
CPCSEA	Committee for the Purpose of Control and Supervision of Experiments on Animals (India)
DMEM	Dulbecco’s Modified Eagle Medium
DMSO	Dimethyl sulfoxide
DPPH	2,2-Diphenyl-1-Picrylhydrazyl
ECM	Extracellular matrix
EDTA	Ethylenediaminetetraacetic Acid
ELISA	Enzyme-Linked Immunosorbent Assay
FBS	Fetal bovine serum
FeCl_3_	Ferric Chloride
FTIR	Fourier-Transform Infrared (Spectroscopy)
GAE	Gallic acid equivalents
GSE	Ginsenoside equivalents
HaCaT	Human Keratinocyte Cell Line (immortalized)
H&E	Hematoxylin and eosin
HO-1	Heme Oxygenase-1 axis
H_2_O_2_	Hydrogen Peroxide
IAEC	Institutional Animal Ethics Committee
IC_50_	Half-maximal inhibitory concentration
IMTECH	Institute of Microbial Technology (CSIR, India)
LC-QTOF-MS	Liquid Chromatography-Quadrupole Time-of-Flight Mass Spectrometr
LC_50_	Lethal concentration for 50% of organisms
LT	*Lobelia trigona/alsinoides* ethanolic extract (as used for the ointment)
MBC	Minimum bactericidal concentration
MFC	Minimum fungicidal concentration
MIC	Minimum inhibitory concentration
MRSA	Methicillin-resistant *Staphylococcus aureus*
MTS	Masson’s trichrome staining
MTCC	Microbial Type Culture Collection
MTT	3-(4,5-Dimethylthiazol-2-yl)-2,5-Diphenyl-2H-Tetrazolium Bromide
NA	Nutrient Agar
NADH	Nicotinamide Adenine Dinucleotide (reduced form)
NBT	Nitro blue tetrazolium
NCCS	National Centre for Cell Science (India)
NF-κB	Nuclear factor kappa-light-chain-enhancer of activated B cells
NMR	Nuclear Magnetic Resonance
Nrf2	Nuclear factor erythroid 2-related factor 2
PBS	Phosphate-buffered saline
PDA	Potato Dextrose Agar
PMS	Phenazine Methosulfate
qRT-PCR	Quantitative Real-Time Reverse Transcription Polymerase Chain Reaction
R^2^	Coefficient of Determination
RE	Rutin equivalents
ROS	Reactive oxygen species
SOB	Simple ointment base
TBA	Thiobarbituric acid
TCA	Trichloroacetic acid
TGF-β	Transforming growth factor-beta
TNAU	Tamil Nadu Agricultural University (India)
UAE	Ursolic acid equivalents
VEGF	Vascular endothelial growth factor
*v*/*v*	Volume per Volume
*w*/*w*	Weight per Weight
