# Supplementary material for: Accelerated Full-Thickness Wound Healing by a Topical Ointment Formulated with Lobelia alsinoides Lam. Ethanolic Extract
Source: Int J Mol Sci. 2025 Nov 1;26(21):10663. doi: 10.3390/ijms262110663 (PMC12608917; doi:10.3390/ijms262110663)
Supplement: Supplementary file 1 [file ijms-26-10663-s001.zip › ijms-3929611-supplementary.pdf]

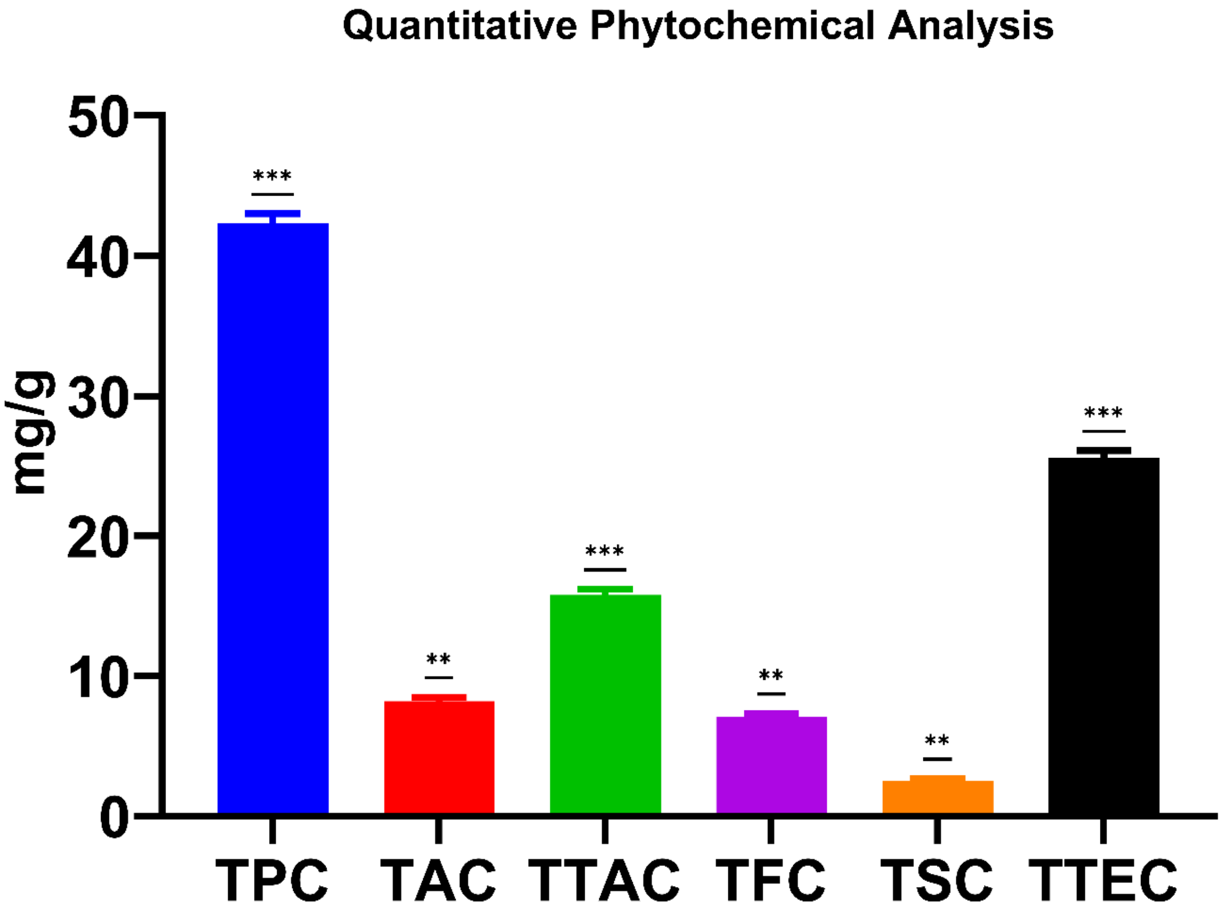

Figure S1. Quantitative phytochemical composition of *Lobelia alsinoides* Lam. ethanolic extract. Data are expressed as mean ± SEM ($n = 3$). Quantitative estimation of major secondary metabolites was carried out using standardized spectrophotometric methods. The concentrations of each phytochemical class were expressed as equivalents of respective reference standards: total phenolic content (TPC) as mg gallic acid equivalents (GAE)/g, total alkaloid content (TAC) as mg atropine equivalents (AE)/g, total tannin content (TTAC) as mg GAE/g, total flavonoid content (TFC) as mg rutin equivalents (RE)/g, total saponin content (TSC) as mg ginsenoside equivalents (GSE)/g, and total terpenoid content (TTEC) as mg ursolic acid equivalents (UAE)/g. All measurements were performed in triplicate. Statistical significance: ** $p < 0.01$, *** $p < 0.001$.

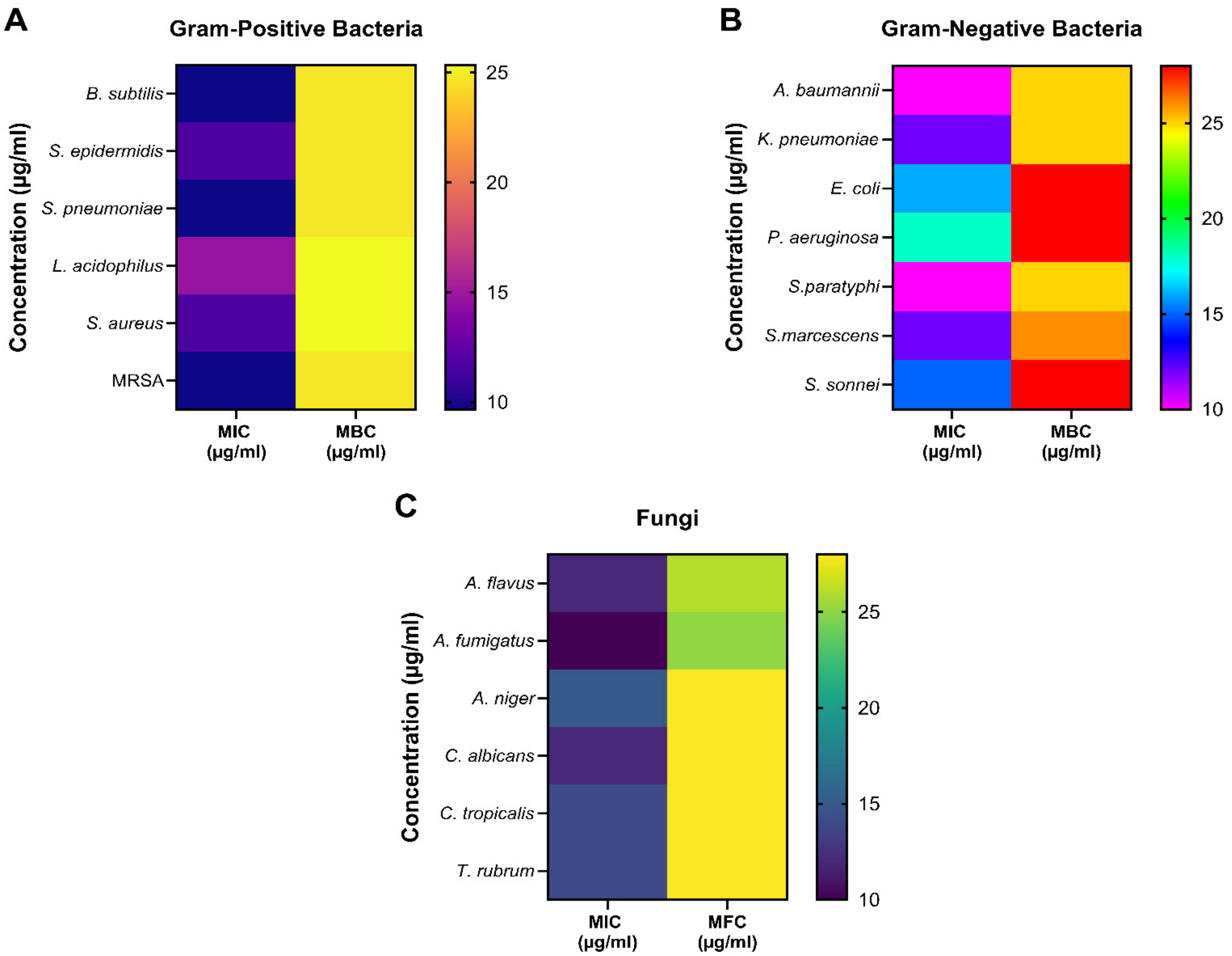

Figure S2. Antimicrobial and antifungal activity of *Lobelia alsinoides* Lam. ethanolic extract expressed as MIC, MBC, and MFC values. Heat maps depict the minimum inhibitory concentration (MIC) and minimum bactericidal concentration (MBC) of the extract against (A) Gram-positive bacteria and (B) Gram-negative bacteria, and the minimum fungicidal concentration (MFC) against (C) fungal pathogens. The colour scale represents concentration gradients (µg/mL), with lower MIC/MBC/MFC values indicating stronger antimicrobial potency.

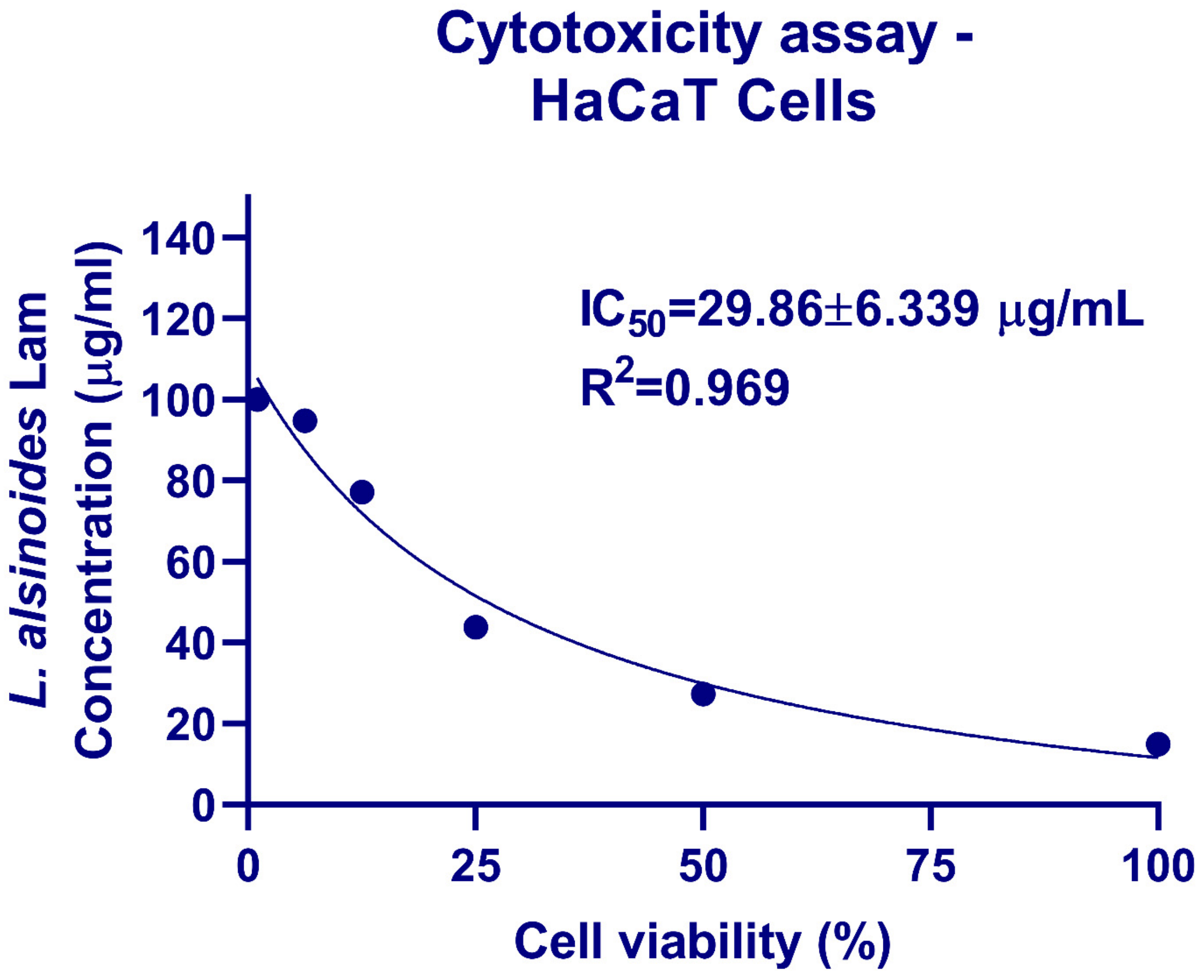

Figure S3. *In vitro* cytotoxicity of *Lobelia alsinoides* Lam. ethanolic extract on HaCaT human keratinocyte cells assessed by the MTT assay. Cells were treated with increasing concentrations of the extract, and viability was expressed as percentage of untreated controls. Cell viability remained above 95% across the tested concentration range, indicating excellent cytocompatibility and supporting the safety of the extract for topical application.

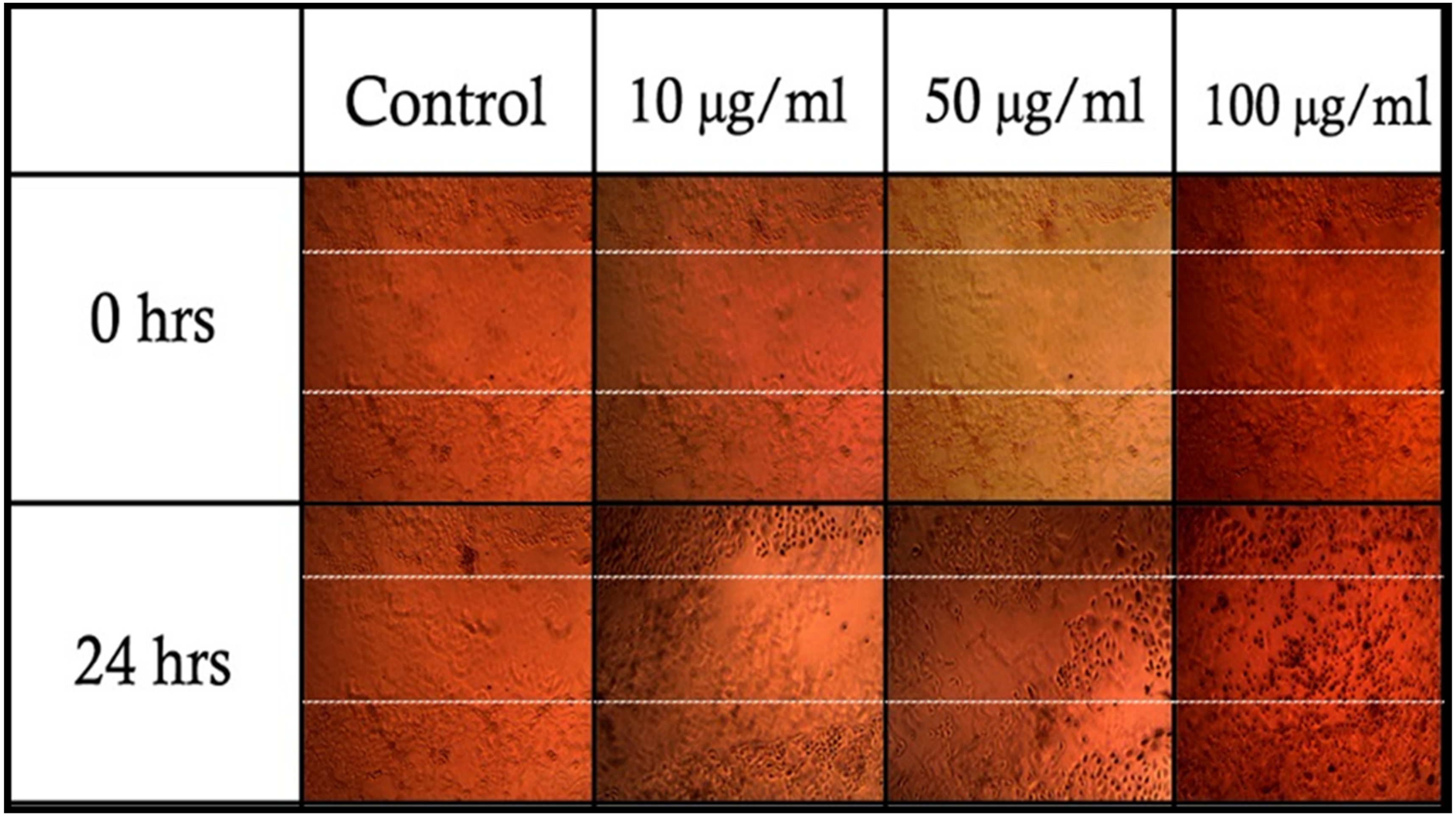

Figure S4. Scratch wound assay demonstrating the pro-migratory effect of *Lobelia alsinoides* Lam. ethanolic extract on HaCaT keratinocytes. Confluent monolayers were scratched and treated with the extract at concentrations of 10, 50, and 100 µg/mL. Representative images were captured at 0 and 24 hours. A clear dose-dependent enhancement of wound closure was observed, with near-complete gap closure at 100 µg/mL, indicating strong wound-healing potential through stimulation of keratinocyte migration. Images were acquired at 10× magnification; scale bar = 100 µm.

Table S1. Nonlinear regression analysis and goodness-of-fit parameters for DPPH, hydroxyl, and superoxide radical scavenging activities of *Lobelia alsinoides* Lam ethanolic extract.

Nonlinear Fit: [Inhibitor] vs. Response (Three Parameters)	DPPH Radical Scavenging Activity	Hydroxyl Radical Scavenging Activity	Superoxide Radical Scavenging Activity
**Best-Fit Values**			
IC_{50} (µg/mL)	15.65	84.78	9.404
$logIC_{50}$	1.195	1.928	0.9733
**95% CI (Profile Likelihood)**			
IC_{50} (µg/mL)	8.967–25.96	46.58–196.3	6.363–13.15
$logIC_{50}$	0.9526–1.414	1.668–2.293	0.8037–1.119
**Goodness of Fit**			
Degrees of Freedom	15	15	15
R^2	0.9694	0.971	0.9884
Sum of Squares	392.1	152.6	186.3
Sy.x	5.113	3.19	3.524

Table S2. Toxicity evaluation of *Lobelia alsinoides* Lam. ethanolic extract.

Toxicity evaluation methods	Time	Ethanolic extract of *Lobelia alsinoides* Lam
**Brine Shrimp Lethality Assay (LC_{50})**	6 hrs	No death
	12 hrs	1.6 mg/ml
	24 hrs	0.75 mg/ml
**Acute Dermal Toxicity (LD_{50})**	14 days	> 2000 mg/kg

Values are expressed as mean ± SEM (n = 6).

Table S3. Statistical analysis of wound healing parameters in rats treated with *Lobelia alsinoides* Lam. ethanolic extract ointments. Significance levels were denoted as: * $p < 0.05$, ** $p < 0.01$, *** $p < 0.001$,

Parameter Analyzed	Wound Contraction	Total Protein	Total Hydroxyproline	Total Hexosamine	Total Uronic acid
**ANOVA summary**					
F	42.26	23.47	20.47	14.77	3.315
P value	<.001	<.001	<.001	<.001	0.047
P value summary	***	***	***	***	*
Significant diff. among means (P < 0.05)?	Yes	Yes	Yes	Yes	Yes
R square	0.8879	0.8149	0.7933	0.7346	0.3833
**Brown-Forsythe test**					
F (DFn, DFd)	0.3752 (3, 16)	0.82 (3, 16)	1.197 (3, 16)	0.06858 (3, 16)	0.9484 (3, 16)
P value	0.772	0.502	0.343	0.976	0.441
P value summary	ns	ns	ns	ns	Ns
Are SDs significantly different (P < 0.05)?	No	No	No	No	No
**Bartlett's test**					
Bartlett's statistic (corrected)	1.591	3.53	7.095	0.1247	8.349
P value	0.661	0.317	0.069	0.989	0.039
P value summary	ns	ns	ns	ns	*
Are SDs significantly different (P < 0.05)?	No	No	No	No	Yes
**Tukey's multiple comparisons test**					
Day 4 vs. Day 8	** (0.001)	*** (<.001)	*** (<.001)	*** (<.001)	*** (<.001)
Day 4 vs. Day 12	*** (<.001)	*** (<.001)	*** (<.001)	*** (<.001)	*** (<.001)
Day 4 vs. Day 16	*** (<.001)	*** (<.001)	*** (<.001)	*** (<.001)	*** (<.001)
Day 8 vs. Day 12	* (0.023)	*** (<.001)	*** (<.001)	*** (<.001)	*** (<.001)
Day 8 vs. Day 16	*** (<.001)	*** (<.001)	*** (<.001)	*** (<.001)	*** (<.001)
Day 12 vs. Day 16	ns	*** (<.001)	*** (<.001)	*** (<.001)	*** (<.001)

Table S4. Biotoxicity analysis of serum biochemical markers in rats following topical application of *Lobelia alsinoides* Lam. ethanolic extract ointments. Parameters of liver and kidney function were measured on Day 16 across experimental groups: untreated control, simple ointment base (SOB), standard (Silverex™), 5% LT, and 10% LT. Values are expressed as mean ± SEM of three independent measurements ($n = 6$).

Bio toxicity assays	Animal groups				
	Control	SOB	Standard (Silverex™)	5% w/w LT	10% w/w LT
Total Bilirubin	0.81 ± 0.13	0.91 ± 0.11	0.52 ± 0.13	0.40 ± 0.24	0.35 ± 0.13
Total protein (gm/dL)	9.31 ± 0.81	8.12 ± 0.12	6.81 ± 0.13	5.2 ± 0.29	4.9 ± 0.22
Aspartate Aminotransferase (AST/SGOT) (U/L)	58.12 ± 4.12	51.84 ± 4.84	43.71 ± 3.52	35.27 ± 2.55	32.18 ± 2.24
Alanine Aminotransferase (ALT/SGPT) (U/L)	47.30 ± 2.43	48.42 ± 0.22	28.33 ± 1.26	23.89 ± 0.43	21.91 ± 0.31
Alkaline phosphatase (U/L)	153.42 ± 3.52	144.11 ± 2.72	124.95 ± 1.84	115.35 ± 1.23	110.12 ± 1.23
Urea (mg/dL)	41.18 ± 1.62	41.22 ± 0.25	32.18 ± 1.33	24.47 ± 0.67	22.27 ± 0.57
Creatinine (mg/dL)	1.32 ± 0.06	1.1 ± 0.12	0.84 ± 0.16	0.67 ± 0.13	0.52 ± 0.15
